# Practical Guidance in Genome-Wide RNA:DNA Triple Helix Prediction

**DOI:** 10.3390/ijms21030830

**Published:** 2020-01-28

**Authors:** Elena Matveishina, Ivan Antonov, Yulia A. Medvedeva

**Affiliations:** 1 Faculty of Bioengineering and Bioinformatics, Lomonosov Moscow State University, 119234 Moscow, Russia; 2 Institute of Bioengineering, Research Center of Biotechnology, Russian Academy of Science, 117312 Moscow, Russia; 3 Department of Biological and Medical Physics, Moscow Institute of Physics and Technology, 141701 Dolgoprudny, Russia; 4 Department of Computational Biology, Vavilov Institute of General Genetics, Russian Academy of Science, 117971 Moscow, Russia

**Keywords:** long noncoding RNA structure, RNA:DNA triple helix

## Abstract

Long noncoding RNAs (lncRNAs) play a key role in many cellular processes including chromatin regulation. To modify chromatin, lncRNAs often interact with DNA in a sequence-specific manner forming RNA:DNA triple helices. Computational tools for triple helix search do not always provide genome-wide predictions of sufficient quality. Here, we used four human lncRNAs (MEG3, DACOR1, TERC and HOTAIR) and their experimentally determined binding regions for evaluating triplex parameters that provide the highest prediction accuracy. Additionally, we combined triplex prediction with the lncRNA secondary structure and demonstrated that considering only single-stranded fragments of lncRNA can further improve DNA-RNA triplexes prediction.

## 1. Introduction

Long noncoding RNAs (lncRNAs) are usually defined as transcripts of more than 200 nt in length and demonstrating no protein-coding capacity. LncRNAs are often lowly expressed and highly tissue-specific as compared to protein-coding genes. These properties of lncRNAs lead to difficulties in the robust detection of their transcription as well as detailed reconstruction of the transcript structure [1,2]. Yet, a combination of several RNA-sequencing techniques improved the detection of lncRNAs [3,4]. Currently, the total number of lncRNAs annotated in the human and mouse genomes is close to the number of protein-coding genes [5,6]. The functional role of the majority of lncRNAs is still unclear. On the other hand, transcription of lncRNAs is regulated [3,7], supporting their functional importance. Indeed, it has been shown that lncRNAs function via surprisingly diverse molecular mechanisms on the transcriptional and posttranscriptional levels (reviewed in [8,9,10]), playing a key role in many cellular processes, including epigenetic regulation of transcription via interaction with chromatin [10]. Being located in the nucleus of mammalian cells [11] lncRNAs often mediate transcription by targeting chromatin-modifying complexes [12,13] or transcription factors (TFs) [14] to specific genomic loci. In addition to protein binding capacity, RNA has the capacity to form hydrogen bonds on both sides of the DNA strand. RNA forms both Watson–Crick and non-Watson–Crick pairs on the Watson–Crick face of DNA strand and Hoogsteen bonds on the other side of DNA strand [15]. As a result, lncRNAs use different molecular mechanisms to bind to the chromatin, including RNA binding to single-stranded DNA regions (known as R-loops) [16], co-transcriptional RNA:RNA interactions based on Watson-Crick pairing [17] and direct RNA:DNA hybridization via triple helices based on Hoogsteen interactions [18,19]. Hoogsteen or reverse Hoogsteen bonds are formed between a single-stranded nucleotide and a purine nucleotide in a double-stranded nucleic acid molecule. An RNA strand becomes parallel or antiparallel to the DNA strand [20]. UC motives in RNA strand tend to form parallel triplexes, AG motives tend to form antiparallel triplexes, while GU motives could form both [20]. Hoogsteen bonds are weaker than Watson-Crick bonds, resulting in Hoogsteen pairing rules being less strict [20].

There are several known cases of lncRNAs involved in chromatin regulation via formation of triple helices with DNA in specific regions. HOTAIR binds to DNA and recruits two chromatin-modifying complexes: PRC2 and LSD1-CoREST, leading to transcription repression in trans [21]. lncRNA ANRIL binds to DNA in cis and recruits PRC1 and PRC2, which in turn represses transcription [21]. LncRNA MEG3 regulates Wnt/β-catenin [22], VEGF [23] and TGF-β [13] pathways by formation of triple helices and in this way attracting PRC2 to target genes [13]. MEG3 ability to form triplexes was validated via different experimental methods [13]. Fendrr recruits PRC2 via RNA:DNA triplex formation and interacts with Trithorax group/Mixed lineage leukemia (TrxG/Mll) complex [12].

Yet, genome-wide prediction of RNA:DNA triplex-based interactions remains a challenging task with a great dependency on triplexes parameters and with a lot of false positive predictions [24]. Single-stranded ribonucleotide chains (including lncRNAs) tend to fold into thermodynamically stable structures [25]. In many cases, the secondary structure of lncRNAs dictates their function (reviewed in detail in [26]). According to the Hoogsteen rules an RNA region cannot form both duplexes and triplexes at the same time. This implies that triplexes can be formed by single-stranded RNA regions only. Indeed, some data indicate that the validated DNA binding domains of the MEG3 lncRNA might correspond to the mainly unpaired RNA regions in the experimentally identified secondary structure model [27]. In this work, we test if adding a predicted secondary structure of lncRNA in the triple helix search increases prediction specificity.

## 2. Results

### 2.1. Fitting Triplex Parameters

**Figure 1:**
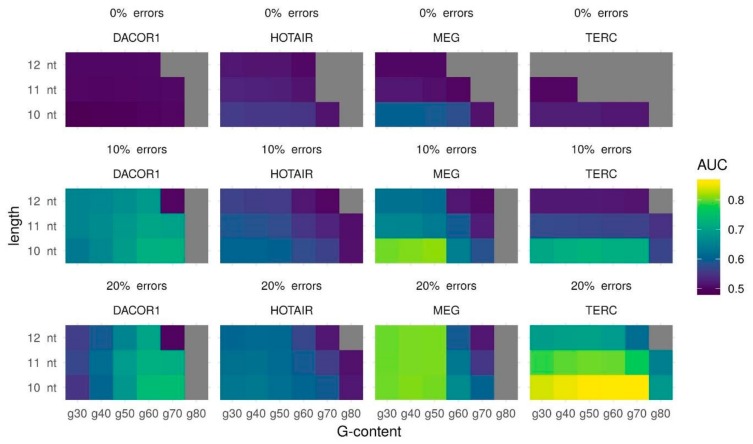
Triplexes predictions for four lncRNAs (TERC, MEG3, DACOR1, HOTAIR) and their experimentally validated target regions with different Triplexator parameters: minimum triplex length, maximum error rate, and minimum G-content. Prediction quality is measured using the AUC (color scheme). Gray color represents the absence of predicted triplexes.

Known cases of lncRNA forming triplexes suggest that actual triple helices may vary in length and number of mismatches. Additionally, enrichment in GA-rich sequences is associated with the formation of stronger triple helices [13]. This makes it reasonable to tune triplexes parameters such as the minimum triplex length, the maximum error-rate, and the minimum G-content to see if there is a combination of the parameters that works best for the majority of lncRNAs with known binding regions. Areas Under the Curve (AUC) for several different parameter combinations are provided in Table S1 and Figure 1. These results clearly suggest that 10 nt as a minimum length of predicted triple helix gives the best AUC in all cases. No errors permitted leads to a very poor prediction quality: AUC close to 0.5 or lack of predicted triplexes. For three out of four lncRNAs (DACOR1, HOTAIR, and TERC) using 20% of the errors leads to the best results. For lncRNA MEG3 both 10% and 20% of the errors give similar results (AUC for 10% errors is 0.0044 higher than AUC for 20% errors). For the sake of uniformity, we suggest that 10 nt and 20% errors are the parameters that are likely to provide the best results. In terms of minimal G-content, lncRNA may be divided into two groups: those that form high (at least 70%) and low (at least 40%) G-content triplexes: TERC and DACOR1 vs. MEG3 and HOTAIR, respectively.

Summing up, we demonstrate that for lncRNAs TERC and DACOR1, the best AUC could be obtained with a minimum length of 10 nt, a maximum error rate of 20%, and a minimum G-content of 70% (AUC = 0.8601 and AUC = 0.7423, respectively), while for MEG3 and HOTAIR, the best AUC could be obtained with a minimum length of 10 nt, a maximum error rate of 20%, and a minimum G-content of 40% (AUC = 0.8078 and AUC = 0.6372, respectively) (Table 1). It should be noted that while a minimum length of 10 bp and a maximum error rate of 20% produce the best AUC for all lncRNAs, the minimum G-content can vary between lncRNAs splitting them into two groups (Table 1). As we see no reason to give any preference to one G-content threshold over another, we suggest to try both sets of parameters.

### 2.2. Secondary Structure Predictions

**Figure 2:**
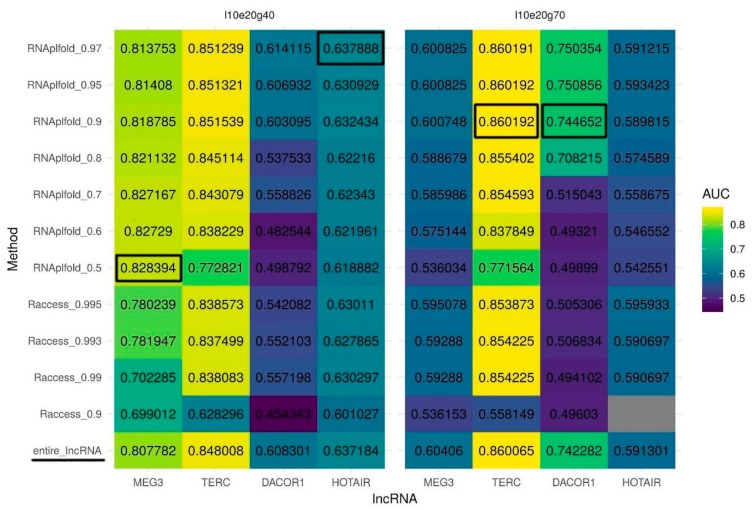
Quality of triplexes predictions with the two sets of parameters. (left panel) The best set of parameters for lncRNAs MEG3 and HOTAIR (l10e20g40: minimum length of 10 bp, maximum error rate of 20%, minimum G-content of 40%, Table 1); (right panel) The best set of parameters for lncRNAs TERC and DACOR1 (l10e20g70: minimum length of 10 bp, maximum error rate of 20%, minimum G-content of 70%, Table 1). Two secondary structure prediction tools were evaluated: RNAplfold and Raccess with a wide range of thresholds. The last row (entire_lncRNA) corresponds to a quality of triplexes prediction with no secondary structure being used. Prediction quality is measured by an AUC (colored scheme). Gray color represents the lack of predicted triplexes.

According to the Hoogsteen rules, only unpaired RNA nucleotides can participate in triplex formation. Thus, we speculate that information about the lncRNA secondary structure may reduce the number of false positive predictions and as a result may increase prediction quality (AUC). To identify unpaired regions within lncRNAs, we test two tools for secondary structure prediction: RNAplfold and Raccess. To run these tools, we used several different thresholds for nucleotide pairing probability and the best sets of Triplexator parameters determined above. If a nucleotide in a lncRNA was predicted to be paired it was masked by the N character and the modified sequence was used for triple helix prediction. Generally, structures predicted with RNAplfold outperform those predicted by Raccess in terms of AUC for triple helix predictions. Importantly, we observed that some parameters of RNA secondary structure prediction result in a huge decrease in the AUC values compared to the original unmasked sequence (Supplementary Figure S1). Yet, for MEG3, using only unpaired (single-stranded) regions of the lncRNA increases the AUC for triplex predictions for structures predicted by RNAplfold but not by Raccess (Figure 2, left panel). Surprisingly, to achieve the best quality of prediction for MEG3 we have to set the probability of pairing as low as 0.5 (RNAplfold). For other lncRNAs usage of solely single-stranded sequences predicted by RNAplfold but not by Racceess also improves the quality of triplexes prediction in most of the cases. For DACOR1 and TERC (Figure 2, right panel), the highest improvement of triplex prediction is achieved when a threshold for a probability of pairing is set to 0.95, while for HOTAIR (Figure 2, left panel), the best results are achieved with a pairing probability threshold of 0.97.

RNAplfold also outperforms Raccess based on partial AUC at 10% FPR in almost all the cases (Supplementary Figure S2) (for the reference, partial AUC at 10% FPR sequences for a random classifier is 0.5%). Partial AUC for MEG3 (the best parameters determined above: l10e20g40, RNAplfold pairing probability 0.5) is increased dramatically (from 4.76% to 6.05%) as compared to the increase in full AUC suggesting an improvement in the prediction of triplexes with the highest normalized frequencies (t_pot_). Partial AUC for DACOR1 (the best parameters determined above: l10e20g70, RNAplfold pairing probability 0.95) is also increased (from 2.7% to 3.55%), supporting the biggest improvement for the sequences with the highest normalized frequencies of predicted triplexes. Partial AUC for TERC increases only slightly (from 3.696% to 3.704%). Partial AUC for HOTAIR is not increased with the previously determined parameters (l10e20g40, RNAplfold pairing probability 0.97), while it shows a slight increase (from 1.01% to 1.07%) when the RNAplfold pairing probability threshold is set to 0.8.

Summing up, we demonstrate that RNAplfold outperforms Raccess in terms of AUC and partial AUC for 10% FPR triplexes predicted. Three thresholds for probability of pairing lead to best results: 0.5 for MEG3, 0.95 for DACOR1 and TERC, and 0.97 for HOTAIR.

### 2.3. Triplex Prediction Using Secondary Structure

**Figure 3:**
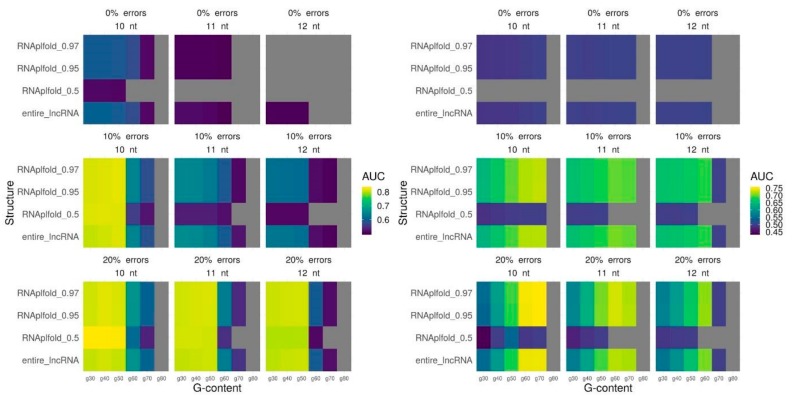
Triplexes prediction quality with different triplexes parameters: minimum length (nt), maximum error-rate, minimum G-content for the entire lncRNA and three RNAplfold thresholds for selection of single-stranded fragments. Prediction quality is measured by AUC (colored scheme), gray color means no triplexes predicted. MEG3 lncRNA (left panel) DACOR1 lncRNA (right panel).

**Figure 4:**
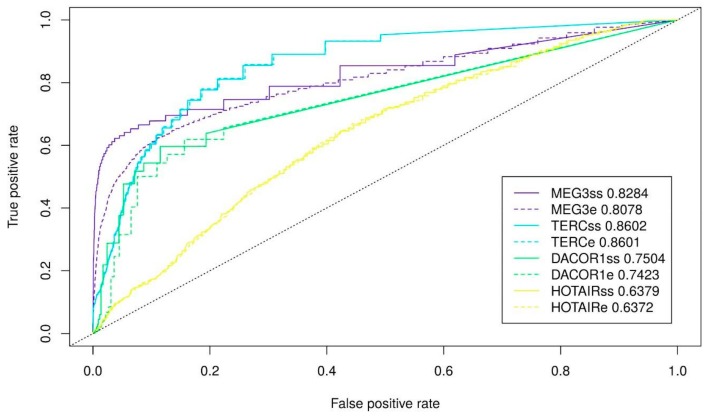
ROC-curves for the best cases of triplexes prediction and corresponding AUC. MEG3ss, and MEG3e stand for single-stranded fragments and the entire MEG3, respectively (l10e20g40, RNAplfold pairing probability 0.5). TERCss and TERCe stand for single-stranded fragments and the entire TERC, respectively (l10e20g70, RNAplfold pairing probability 0.95). DACOR1ss and DACOR1e stand for single-stranded fragments and the entire of DACOR1, respectively (l10e20g70, RNAplfold pairing probability 0.95). HOTAIRss and HOTAIRe stand for single-stranded fragments and the entire HOTAIR, respectively (l10e20g40, RNAplfold pairing probability 0.97).

Finally, we fit the parameters for triplex prediction in combination with the probability of nucleotide pairing based on the secondary structure for all lncRNAs. We vary a minimum length, maximum error-rate, and minimum G-content for predicted triplexes and used three different RNAplfold thresholds (0.5, 0.95, 0.97) that performed best for the whole lncRNAs. In the majority of the cases, parameters performed the best for the entire lncRNA appears to be the best parameters also when only single-stranded lncRNA regions are used for triplex prediction: a minimum length of 10 nt and a minimum error-rate of 20% (Figure 3, Supplementary Figure S3). A tendency of a lncRNA to form a low G-content (40%) or high G-content (70%) triplexes also retains (Figure 3, Supplementary Figure S3). To conclude, we achieve the best quality of triplexes prediction using the following set of parameters: for MEG3 (l10e20g40 and RNAplfold pairing probability of 0.5), for TERC (l10e20g70 and RNAplfold pairing probability of 0.95), for DACOR1 (l10e20g40 and RNAplfold pairing probability of 0.95), and for HOTAIR (l10e20g70 and RNAplfold pairing probability of 0.97). It should be noted that the most significant improvement is achieved for MEG3. The ROC (receiver operating characteristic)-curves with the best prediction quality are provided on Figure 4.

### 2.4. DNA Binding Domains (DBDs) and Single-Stranded Fragments

**Figure 5:**
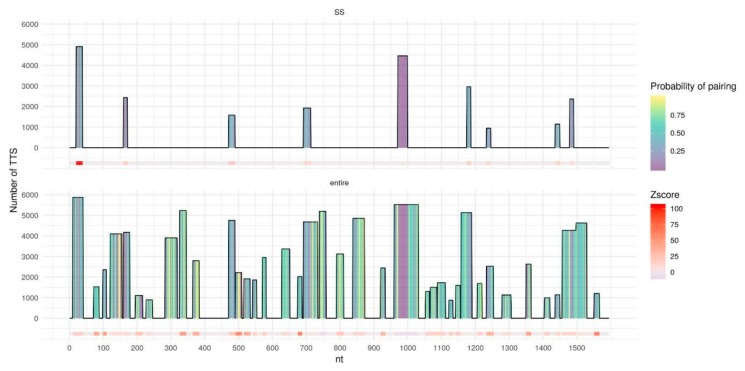
DNA binding domains (DBDs) of the entire MEG3 (bottom) and of single-stranded fragments of MEG3 (top), predicted by TDF with MEG3 best parameters (l10e20g40). The horizontal axis represents MEG3 in length, columns represent DBDs with height corresponding to the number of DNA peaks (Triplex target sites, TTS) with predicted triplexes for the particular DBD. DBDs columns are filled with color by the probability of pairing for each nucleotide predicted by RNAplfold. DBDs in the MEG3 sequence are colored by z-score for a particular DBD calculated by TDF based on random samplings from the human genome; z-score = 0 corresponds to no DBDs found.

**Figure 6:**
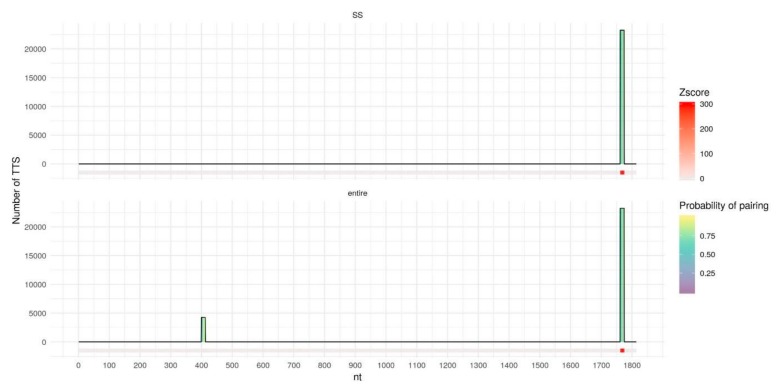
DNA binding domains (DBDs) of the entire DACOR1 (bottom) and of single-stranded fragments of DACOR1 (top), predicted by TDF with DACOR1 best parameters (l10e20g70). The horizontal axis represents DACOR1 in length, columns represent DBDs with height corresponding to the number of DNA peaks (Triplex target sites, TTS) with predicted triplexes for the particular DBD. DBDs columns are filled with color by the probability of pairing for each nucleotide predicted by RNAplfold. DBDs in the DACOR1 sequence are colored by z-score for a particular DBD calculated by TDF based on 10,000 random samplings from the human genome; z-score = 0 corresponds to no DBDs found.

We further investigate why the usage of the secondary structure is the most beneficial in the case of MEG3. To do so we detect DNA binding domains (DBDs) - fragments of lncRNAs that form the majority of triplexes with the target DNA regions - using Triplex Domain Finder (TDF) [28] both for entire lncRNAs and for single-stranded fragments. In the case of the entire MEG3 (Figure 5, bottom) TDF found a lot of long DBDs that form a significant number of triplexes with the target DNA regions. Yet, many of these DBDs contain nucleotides with a relatively high probability of pairing, thus, considering only single-stranded fragments (Figure 5, top) leads to removal or shrinkage of such DBDs. Remaining DBDs form triplexes with a lot of experimentally validated RNA:DNA interacting regions. Two of the previously reported DBDs: 20–38 nt and 971–999 nt [13,28] increase their z-score. In the case of MEG3, usage of the secondary structure reduces false positive predictions leading to an improvement of the overall prediction quality. In the case of DACOR1 (Figure 6), only two DBDs are detected for the entire lncRNA. For a single-stranded variant of lncRNA, the DBD with the low number of predicted triplexes is removed, presumably reducing the number of false positives. For the entire TERC and HOTAIR (Supplementary Figures S4 and S5) several DBDs are detected and the majority of them are kept intact or slightly shortened if only a single-stranded RNA is considered. As a result, only a very moderate increase in AUC is observed.

## 3. Discussion

Prediction of triple helix structures genome-wide is a challenging task [24]. Although Triplexator [29] outperforms an alternative approach provided by LongTarget [30], the prediction quality is still to be improved [24]. Recently, a high accuracy model for triplex prediction based on a neuronal network has been proposed [31]. However, it requires data on experimental lncRNA binding for training which is available only for a small number of lncRNAs.

Computational genome-wide prediction of triplexes formed by a particular lncRNA is complicated by the presence of universal triplex target sites (TTS) - regions that are capable of triplex formation with almost any expressed lncRNA [32]. Similarly, we found universal DNA binding domains (DBDs). The most significant one is located at the 3′ end of HOTAIR and forms even more triplexes with random DNA fragments than with the HOTAIR target regions obtained in the ChOP-seq experiment (Table S2). Additionally, it has been shown that HOTAIR can form not only triplexes but also duplexes with single-stranded DNA [17]. We speculate that having two different mechanisms of binding may contribute to the low quality of triplex prediction in the case of HOTAIR.

Interestingly, to increase the quality of MEG3 triplex prediction, we had to use a relatively low threshold for base pairing in RNAplfold. It should be noticed that the entire MEG3 has lots of DBDs (and lots of triplex forming oligos (TFOs) - fragments that potentially form triplexes with any possible DNA – that can be obtained from Triplexator (Table S3). The majority of these DBDs cover only a small fraction of MEG3 target sequences. It seems unlikely that all these DBDs or TFOs represent real triplexes suggesting the need to use stricter RNAplfold base pairing threshold (0.5). While DACOR1 and TERC have only a few TFOs (or DBDs) (Table S3) when the best triplex parameters are used (Table 1) and these DBDs are present in many target regions, suggesting that those DBDs are more likely to be functional. Since HOTAIR has two mechanisms of binding, we do not recommend to base any conclusions on the results of this lncRNA.

It should be noted that our approach has several limitations. First, experimental techniques such as ChOP-seq and ChIRP-seq detect only the regions of DNA that interact with a particular lncRNA but do not uncover the interaction mechanisms. For several particular lncRNA:DNA triplex formation has been validated experimentally in vitro [13], but it is difficult to scale this approach to genome- and transcriptome-wide levels. Since ChOP-seq and ChIRP-seq do not uncover the interaction mechanisms of lncRNA and DNA regions, we cannot be absolutely sure that all experimentally detected RNA-DNA interacting regions indeed form triplexes. To overcome this limitation, a new experimental approach that uncovers only interactions via triple helices has been proposed [33]. Yet, this approach is capable only of detecting a pool of DNA-interacting RNAs and vice versa, a pool of RNA-interacting DNA fragments but not the DNA-RNA interacting pairs. To the best of our knowledge, an experimental methods of detecting DNA-RNA triple helices genome- and transcriptome-wide are yet to be developed.

The second limitation of our approach is that Triplexator takes into account only Hoogsteen pairing rules summing up the number of nucleotides that are involved into triplex formation. Yet, other factors could significantly affect triplex fprmation (for example, the G-C nucleotides form stronger triplexes as compared to A-T nucleotides [34]), and therefore, should also be considered in the model.

Third, in this work, we used predicted, rather than experimentally validated RNA secondary structures. Unfortunately, for the majority of lncRNAs for which RNA-DNA binding data is available, experimentally validated structures have not been reported. Moreover, it is known that lncRNA structures are dynamic and adopt multiple conformations; for example, several secondary structures that have been experimentally detected are known for the A-repeat section of lncRNA Xist [35]. Multiple bioinformatic tools for RNA structure prediction have been developed, yet their results differ dramatically and it is not always clear how to choose a predicted structure for a particular RNA. RNAsubopt tool [36] calculates suboptimal secondary RNA structures that might help to find regions that are functional and therefore unpaired in all suboptimal structures. Alternatively, only one suboptimal structure may form a triplex. At this stage, further research is needed to determine a strategy for incorporation of suboptimal structures into the model. Considering dynamic folding and unfolding of different RNA structures (using, for example, thermodynamic constraints on structure folding) might also improve the quality of triplex predictions. Yet, to the best of our knowledge, tools for prediction of RNA structure dynamics have not been developed. Increasing the accuracy of RNA structure prediction, consideration of multiple conformations and RNA structure dynamic could potentially benefit the triplex prediction model.

Fourth, experimental data on RNA-DNA binding is currently available for only a few lncRNAs. Additional information on genome-wide lncRNA binding will provide the possibility to re-evaluate our model and to improve RNA-DNA triplex prediction accuracy.

Although the methods for experimental detection of genome and transcriptome-wide RNA-DNA interactions are being developed (GRID-seq [37], MARGI [38], RADICL-seq [39]), they have limited capacity of detection interactions for low expressed RNAs. Due to the finite sequencing depth mostly contacts between DNA and nascent RNA as well as contacts for RNAs with high expression are detected [24]. High quality computational predictions of RNA-DNA interactions may improve the detection of interactions of low-expressed RNAs. Genome- and transcriptome-wide prediction of high confidence triple helices could help selecting RNA-DNA pairs for small-scale experimental validation with electrophoretic mobility shift assay (EMSA), immunostaining with anti-triplex antibody and other methods. Being experimentally validated particular RNA-DNA interactions may contribute to understanding the mechanisms of human diseases, since, for example, MEG3 and HOTAIR are associated with various cancers (gastric cancer [40], hepatocellular carcinoma [41], cervical cancer [42], etc.).

In this work we focused on the triplexes formed by lncRNA and DNA but they are not the only molecules that can form triplexes. There are several reported cases of triplex formation between mRNA and miRNA (for example, E2F1 mRNA with miR-205–5p and miR-342–3p [43]). miRNAs are also capable to form triplexes with DNA [44], as well as DNA can form triplexes with itself [45]. As Triplexator predicts forming Hoogsteen or reverse Hoogsteen nucleotides interactions we do not see any reason for using it only for lncRNA and DNA. The only constraint is that nucleotide sequence must be at least 10 nt in length.

Summing up, we believe that relaxed parameters for triplex prediction as well as usage of only single-stranded RNA regions and exclusion of regions with an extremely high probability of pairing may improve prediction quality.

## 4. Materials and Methods

### 4.1. lncRNA Selection and Experimental Data on lncRNA Binding

For this analysis, we used four lncRNAs—MEG3 (ENST00000451743.6), TERC (ENST00000363312.1), DACOR1 (TCONS_00023265) and HOTAIR (ENST00000424518.5) - for which triplex formation was experimentally validated as well as data of genome-wide binding was available. Triplexes formation was validated for MEG3 in vitro with electrophoretic mobility shift assay (EMSA), circular dichroism (CD) spectroscopy, cell transfection with biotin-labelled MEG3 triplex-forming oligos (TFO) and in vivo with immunostaining with anti-triplex antibody [13]. HOTAIR triplexes formation was validated via electrophoretic mobility shift assay (EMSA) [46]. TERC ability to form triplexes in vitro was observed via EMSA and melting temperature [47]. HOTAIR, TERC and DACOR1 lncRNAs were also used for evaluating deep learning mode for triplexes prediction [31].

For all lncRNAs, the binding regions (ChIRP-seq and ChOP-seq peaks) were obtained from a corresponding paper (Table 2) and converted to hg38 using liftOver if needed. We used detected peaks for each lncRNA as true positives and random sequences of the same median length from hg38 obtained by bedtools as true negatives.

### 4.2. LncRNA Secondary Structure Prediction

For each lncRNA, we predicted a secondary structure reflecting nucleotides state as being paired or unpaired using Raccess [50] and RNAplfold [51]. Raccess provides energy of pairing (kcal/mol) for a particular nucleotide. The probability of pairing can be calculated using the formula $P=exp\left(-E/RT\right)$, where $RT=0.61633008\left[kcal/mol\right]$. RNAplfold provides a probability of pairing for each nucleotide pair. A nucleotide is considered as paired if it is paired with at least one other nucleotide with a probability above a given threshold. For further predictions paired nucleotides were masked as “N” in the sequence of the particular lncRNA to simulate their inability to bind DNA.

### 4.3. Triple Helix Prediction

We used Triplexator [29] and TDF [28] command-line tools for RNA:DNA triplex prediction. LncRNA and DNA sequences in FASTA format were provided as input. We fitted several triplex parameters such as: minimum triplex length, maximum error-rate, G-content. We do not disregard repeat and low-complex regions as suggested by default settings of Triplexator. For Triplexator predictions, we used a t_pot_ as a measure of triplex prediction quality. It reflects a number of predicted triplexes for each RNA:DNA pair normalized by DNA and lncRNA sequence length. For each lncRNA, TDF provides DNA binding Domains (DBDs), exact regions that form triplexes, their p-value, and z-score, calculated based on 10,000 random samplings from the human genome.

### 4.4. ROC-Curves and AUC

To estimate the quality of predictions we used ROC-curves and AUC measures. We ranked ChIRP-seq and ChOP-seq peaks as well as random background sequences based on t_pot_ considering peaks with no triplexes predicted, constructed ROC-curves and calculated AUC using ROCR package [52]. We also investigated the sequences with the highest normalized frequencies of predicted triplexes (highest t_pot_) using partial AUC for 10% of FPR.

## 5. Conclusions

To conclude, triplexes prediction quality dramatically depends on triplexes parameters. To achieve the highest accuracy, we suggest to use two sets of Triplexator parameters: minimum length of potential triplexes of 10 nt, maximum error-rate of 20% and minimum G-content of 40% or 70%. Additionally, we recommend to mask RNA regions with the highest probability of pairing (0.95–0.97) in a lncRNA that has a few potential TFOs. Yet, if the number of TFO is high, as low as 0.5 probability of pairing may be beneficial.

## Figures and Tables

**Table 1:** Triplexator parameters that generated the best results for each of the four analyzed lncRNAs. Overall, we recommend using a minimum length of 10 bp, a maximum error-rate of 20%, and test a minimum G-content of 40% and 70%.

lncRNA	Min Length (nt)	Max Error-Rate (%)	Min G-Content (%)	AUC	Symbol
MEG3	10	20	40	0.8078	l10 e20g40
HOTAIR	10	20	40	0.6372	l10e20g40
TERC	10	20	70	0.8601	l10e20g70
DACOR1	10	20	70	0.7423	l10e20g70

**Table 2:** Summary statistics and data sources for lncRNAs used in this study.

lncRNA	lncRNA ID	lncRNA Length	Number of DNA Peaks	Median Peaks Length	Method	Ref
MEG3	ENST00000451743.6	1595 nt	6798	400 nt	ChOP-seq	[13]
TERC	ENST00000363312.1	451 nt	2198	756 nt	ChIRP-seq	[48]
DACOR1	TCONS_00023265	1814 nt	40213	279 nt	ChIRP-seq	[49]
HOTAIR	ENST00000424518.5	2421 nt	832	678 nt	ChIRP-seq	[48]

## Supplementary Materials

Supplementary materials can be found at https://www.mdpi.com/1422-0067/21/3/830/s1.
