# Supplementary figures and images for: Practical Guidance in Genome-Wide RNA:DNA Triple Helix Prediction
Source: Int J Mol Sci. 2020 Jan 28;21(3):830. doi: 10.3390/ijms21030830 (PMC7037363; doi:10.3390/ijms21030830)

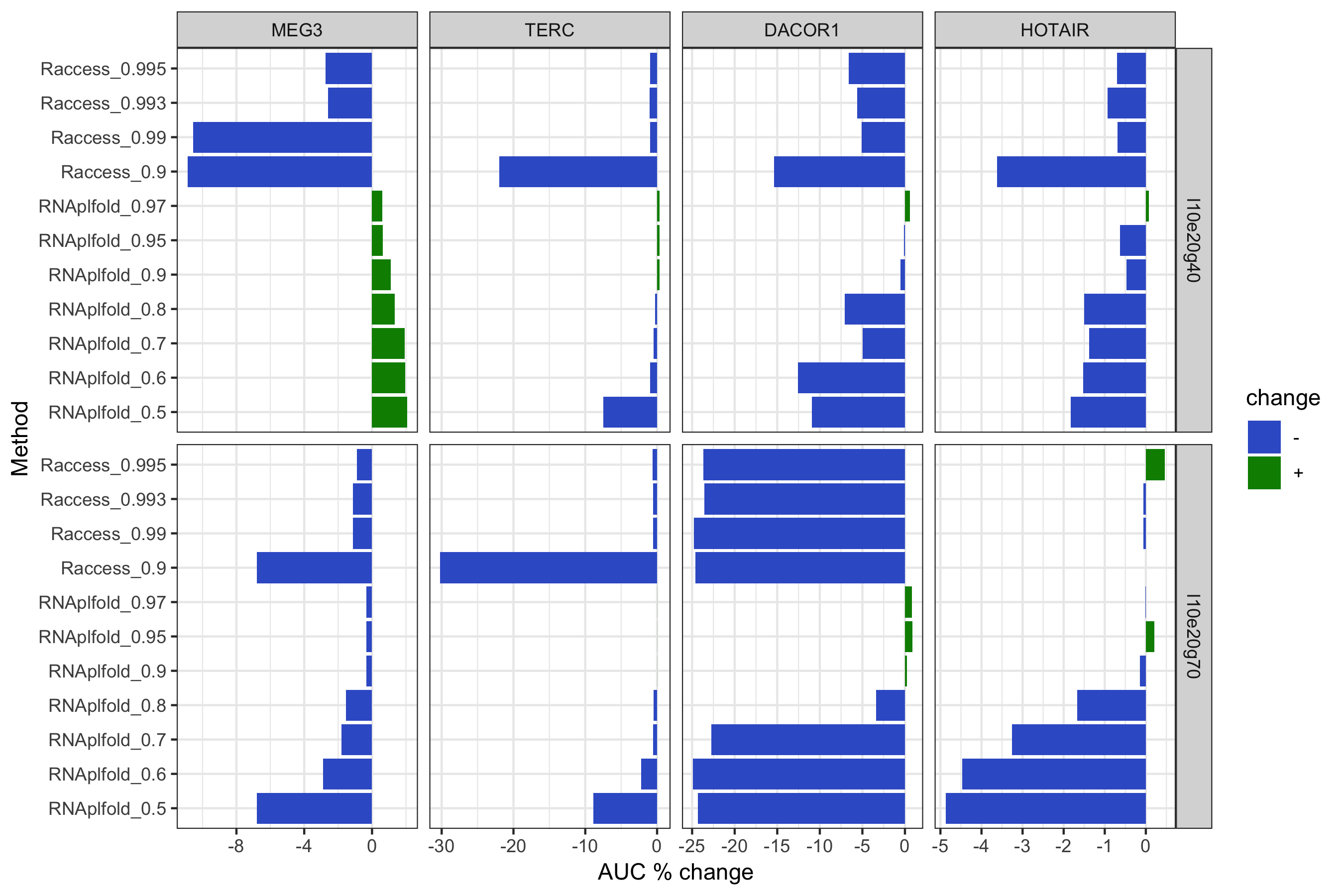

Supplement: Supplementary file 1 [file ijms-21-00830-s001.zip › supplementary/figS1.png]

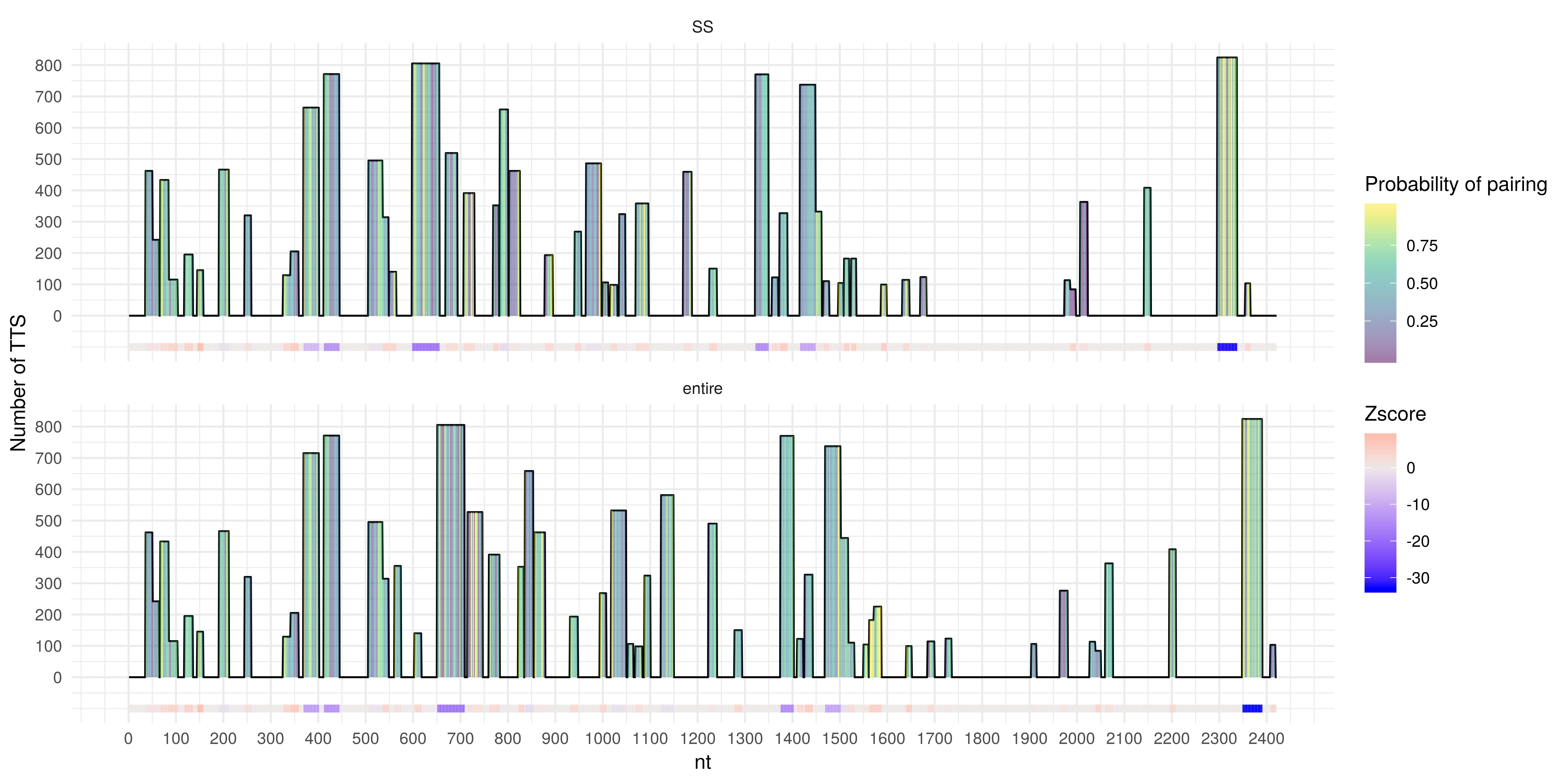

Supplement: Supplementary file 1 [file ijms-21-00830-s001.zip › supplementary/figS5.jpg]
